# Opinions and clinical practice of functional movement disorders: a nationwide survey of clinicians in China

**DOI:** 10.1186/s12883-021-02474-4

**Published:** 2021-11-09

**Authors:** Xin-Yi Xie, Guo-zhen Lin, Qiang Huang, Chun-Bo Li, Mark Hallett, Valerie Voon, Ru-jing Ren, Sheng-di Chen, Gang Wang

**Affiliations:** 1 Department of Neurology and Institute of Neurology, Ruijin Hospital, Shanghai Jiao Tong University School of Medicine, No.197, Ruijin Second Road, Huangpu District, Shanghai, China; 2 Department of Psychiatry, Ruijin Hospital, Shanghai Jiao Tong University School of Medicine, Shanghai, China; 3 Shanghai Key Laboratory of Psychotic Disorders, Shanghai Mental Health Center, Shanghai Jiao Tong University School of Medicine, Shanghai, China; 4 Human Motor Control Section, Medical Neurology Branch, National Institute of Neurological Disorders and Stroke, National Institutes of Health, Bethesda, MD USA; 5 Department of Psychiatry, University of Cambridge, Cambridge, UK

**Keywords:** Functional movement disorders, Psychogenic movement disorders, Survey

## Abstract

**Background:**

There is rare reports about opinions and clinical practice of functional movement disorders (FMD) in China. The present survey aimed to investigate the views of FMD in Chinese clinicians.

**Methods:**

The Chinese version survey of FMD were conducted in nationwide practitioners by means of an online questionnaire.

**Results:**

Four hundred and thirty-four Chinese clinicians completed a 21-item questionnaire probing diagnostic and management issues in FMD. More than 80% of respondents considered that atypical movement disorder, multiple somatizations, and emotional disturbance were essential or absolutely necessary for clinically definite diagnosis of FMD. About three quarters of respondents requested standard neurological investigations to rule out organic causes. Over half believed that prior diagnosis of an organic disorder (59.9%), lack of associated non-physiologic deficits (51.8%), and evidence of physical injury (50.0%) were ‘very influential’ or ‘extremely influential’ for a non-FMD diagnosis. The majority (77.4%) of the respondents may refer patients to a neuropsychiatrist or psychiatrist experienced in FMD, followed by psychologist or psychotherapist experienced in FMD (53.2%). However, lack of guidelines, physician knowledge, and training often limited clinicians’ ability in managing patients with FMD. Early diagnosis of FMD, identification and management of concurrent psychiatric disorder, and acceptance of the diagnosis by the patient were considered most important for predicting a favorable prognosis.

**Conclusions:**

Opinions and clinical practice of Chinese practitioners not only varied among Chinese neurologists, but also differed from international peers. Combined efforts are needed to promote related research and establish practice guidelines in China in the future.

**Supplementary Information:**

The online version contains supplementary material available at 10.1186/s12883-021-02474-4.

## Backgrounds

Functional movement disorders (FMD), also known as psychogenic movement disorders (PMD) and conversion disorders [1], are involuntary movements often attributed to psychological causes [2]. In the past, the term ‘hysteria’ was often used to describe these disorders. FMD are a common source of disability, and specific prevalence of FMD remains unclear so far. Females (60–75%) are more affected than males except for specific subtypes (e.g., functional myoclonus) [3]. Neurobiological mechanisms of FMD are not so clear but abnormalities of attentional focus, belief and expectations, and sense of agency might be involved [4]. Furthermore, overactivity of the limbic system and increased connections to the motor system play a role in pathophysiology of FMD as well [5]. Even though there are an increasing number of positive diagnostic criteria, the diagnosis of FMD is still challenging in the present. The Diagnostic and Statistical Manual of Mental Disorders (DSM)-5 indicates that emphasis should be put on positive clinical characteristics rather than related psychological factors [6]. Previously, two investigations of opinions and clinical practice of FMD at an interval of one decade (2008 and 2018 respectively) were performed for members of the International Parkinson and Movement Disorder Society (MDS). Interesting changes of attitudes and management of FMDs have been found, but gaps remained in access to diagnosis and treatment [7, 8]. However, there is rarely known about opinions and clinical practice of FMDs by Chinese clinicians. The present survey aimed to investigate the views of FMD in Chinese clinicians, predominately neurologists and movement disorders specialists, and reveal the current status concerning FMDs in the clinical setting in China.

## Methods

### FMD questionnaire

The Chinese version of a 21-item FMD questionnaire (Additional file 1) was authorized and adapted from the recent MDS version [8]. A group of experts were served as external reviewers to ensure content and face validity of the survey (8 academic neurologists with expertise in movement disorders and 1 academic psychiatrist were included. Detailed information about affiliations was listed in Table S1). After multiple reviews of these experts and modifications based on suggestions, an online tool (www.wjx.cn) was used to create and issue the questionnaire. URL address and QR code were shared to target clinicians via Wechat and the online survey was available for 2 weeks. Only practitioners who may be exposed to FMD were asked to complete the questionnaire. Participants were asked to complete the questionnaire within 2 weeks.

### Statistical analysis

SPSS 22.0 was used to perform statistical analysis on downloaded data. For descriptive analysis, Chi-Square tests were used in comparison of frequencies and proportions. Average ratings were calculated in Likert scale data. Spearman correlation analysis was performed within rating questions. *P* < 0.05 was considered as statistically significant.

## Results

### Demographics

A total of 434 respondents accomplished the anonymous questionnaire within 2 weeks. Males made up 47.5%, and respondents over age of 45 years old accounted for nearly a half. They were from 29 provinces/ municipalities / autonomous regions in China and demographic information can be found in Fig. S1. Approximately 90% of all respondents were from departments of Neurology or Movement Disorders (MDs) Clinics, and over half practiced less than 5 years in MDs subspecialty. Three quarters of the respondents were practiced in Tertiary hospitals. Detailed demographic characteristics were listed in Table 1.

**Table 1:** Demographics and overall practice of all respondents

Descriptions	N	%
Gender		
Male	206	47.5
Female	228	52.5
Age		
25-35y	76	17.5
36-45y	144	33.2
46-55y	172	39.6
56-65y	40	9.2
> 65y	2	0.5
Fellowship training^a^		
None	186	42.9
1y	89	20.5
2y	20	4.6
≥ 3y	139	32.0
Years of practice in MDs^b^		
≤ 5y	266	61.3
6-10y	79	18.2
11-15y	38	8.8
16-20y	19	4.4
≥ 21y	32	7.4
Department of hospital		
Neurology (General)	322	74.2
Movement Disorders	62	14.3
Others^c^	50	11.5
Number of FMD patients seen per month		
< 1	134	30.9
1–3	143	32.9
4–6	51	11.8
7–10	13	3.0
> 11	16	3.7
Uncertain	77	17.7
Number of all MD patients seen per month		
< 30	234	53.9
30–45	74	17.1
46–60	24	5.5
61–80	10	2.3
> 80	26	6.0
Uncertain	66	15.2
Role or responsibility is in assessing FMD		
Provide only a diagnosis	24	5.5
Diagnose and secure expert management	198	45.6
Diagnose and coordinate interdisciplinary long-term management	177	40.8
Diagnose and manage the care personally	35	8.1
Personal preference in taking care of patients with FMD		
Very much look forward to	53	12.2
Somewhat look forward to	86	19.8
Neither looking forward to nor dislike	188	43.3
Somewhat dislike	97	22.4
Very much dislike	10	2.3

^a^Fellowship training: training of the refresher doctors in subspecialty of MDs

^b^number of years engaged in PD outpatient or predominately engaged in diagnosis and treatment of MDs

^c^Other departments (11.5%) included internal medicine (5.0%), psychiatry (3.0%), and rehabilitation or functional neurosurgery (3.5%)

Among the respondents, the majority acknowledged that they assessed no more than 3 FMD patients per month. Only a minority (18.5%) reported more than three FMD patients seen monthly. Meanwhile, a similar proportion (17.7%) were uncertain about the number of FMD patients they assessed. The number of FMD patients correlated with the number of patients with all movement disorders seen in clinic monthly (*r* = 0.248, *p* < 0.01). In addition, respondents who experienced fellowship training in movement disorders or with longer years in practice reported more FMD patients seen per month (Table 2). When it comes to their responsibility of assessing patients with FMD, the substantial majority (94.5%) believed that their role was not only just to provide a diagnosis, but also included coordinate interdisciplinary management or personal management.

**Table 2:** Fellowship training and years of practice in MDs have an influence on number of FMD patients assessed per month

	FMD patients seen per month (others)	More than 3 FMD patients seen per month	*P* value
N	354	80	
Length of fellowship training			
none	160 (45.2%)	26 (32.5%)	X^2^ = 4.296 *P* = 0.038
1–3 years or longer	194 (54.8%)	54 (67.5%)	
Years of practice in MDs subspecialty			
< 5 years	235 (66.4%)	31 (38.8%)	X^2^ = 21.003 *P* = 0.000
6–21 years or longer	119 (33.6%)	49 (61.3%)	

### Reaching the diagnosis

In this present survey, more than 80% of respondents considered that incongruent movement disorder, multiple somatizations, and emotional disturbance were essential or absolutely necessary for a clinically definite diagnosis of FMD (Fig. S2). Respondents tended to use suggestion to document and diagnose FMD more than placebo (often and always: 29.5% versus 17.7%) (Fig. S3).

**Fig. 1:**
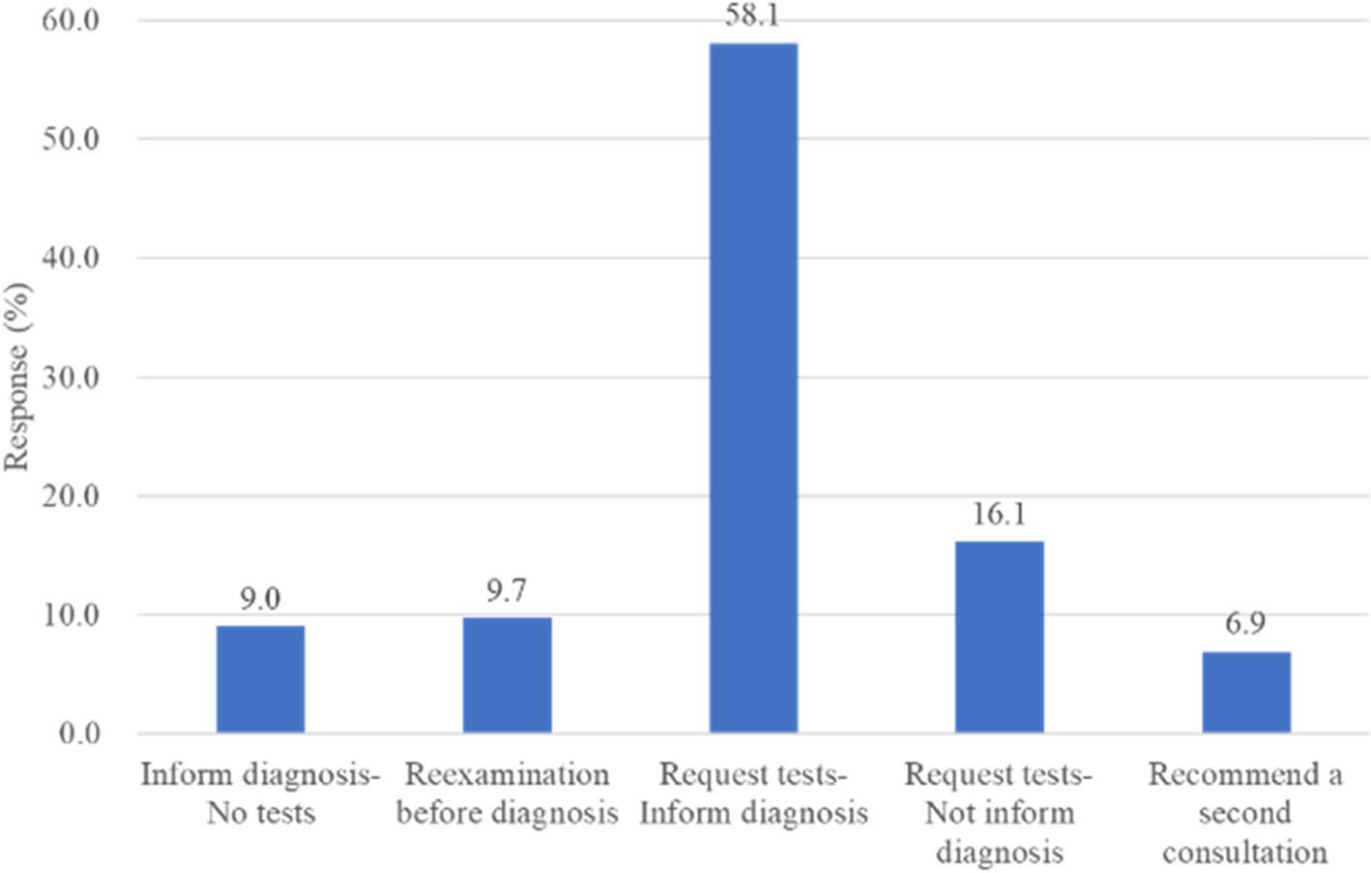
Approach to delivering the diagnosis in clinically definite FMD

In the case of unequivocal clinical features, about three quarters of respondents requested standard neurological investigations with the aim to rule out organic causes. About half (58.1%) requested neurological investigations after which they informed the patients about the diagnosis of FMD, while others (16.1%) did not inform the patient of FMD diagnosis or psychogenic causation. Only 9% of the respondents were confident about making diagnosis (They directly informed the patient about the diagnosis in initial assessment without requesting neurological investigations) (Fig. 1). Approximately 10% responded that they have no access to electrophysiology testing or deemed it useless in diagnosis of FMD. Majority of the rest respondents use the testing in uncertain cases (clinical examination alone is insufficient) or clinically definite FMD cases to confirm diagnosis. The other 16% apply electrophysiology testing in all cases. About two thirds of respondents use testing results sometimes (35.5%), often (24.7%), and always (4.4%) to explain the diagnosis to the patient.

Some of the respondents reported that a potential comorbid organic neurologic disorder was discovered in diagnosed FMD patients sometimes (35.9%) or frequently (1.8%). Meanwhile, more than half of all respondents worried about potentially ignorance of another organic disorder in patients with FMD.

### Predictors for diagnosis other than FMD

**Fig. 2:**
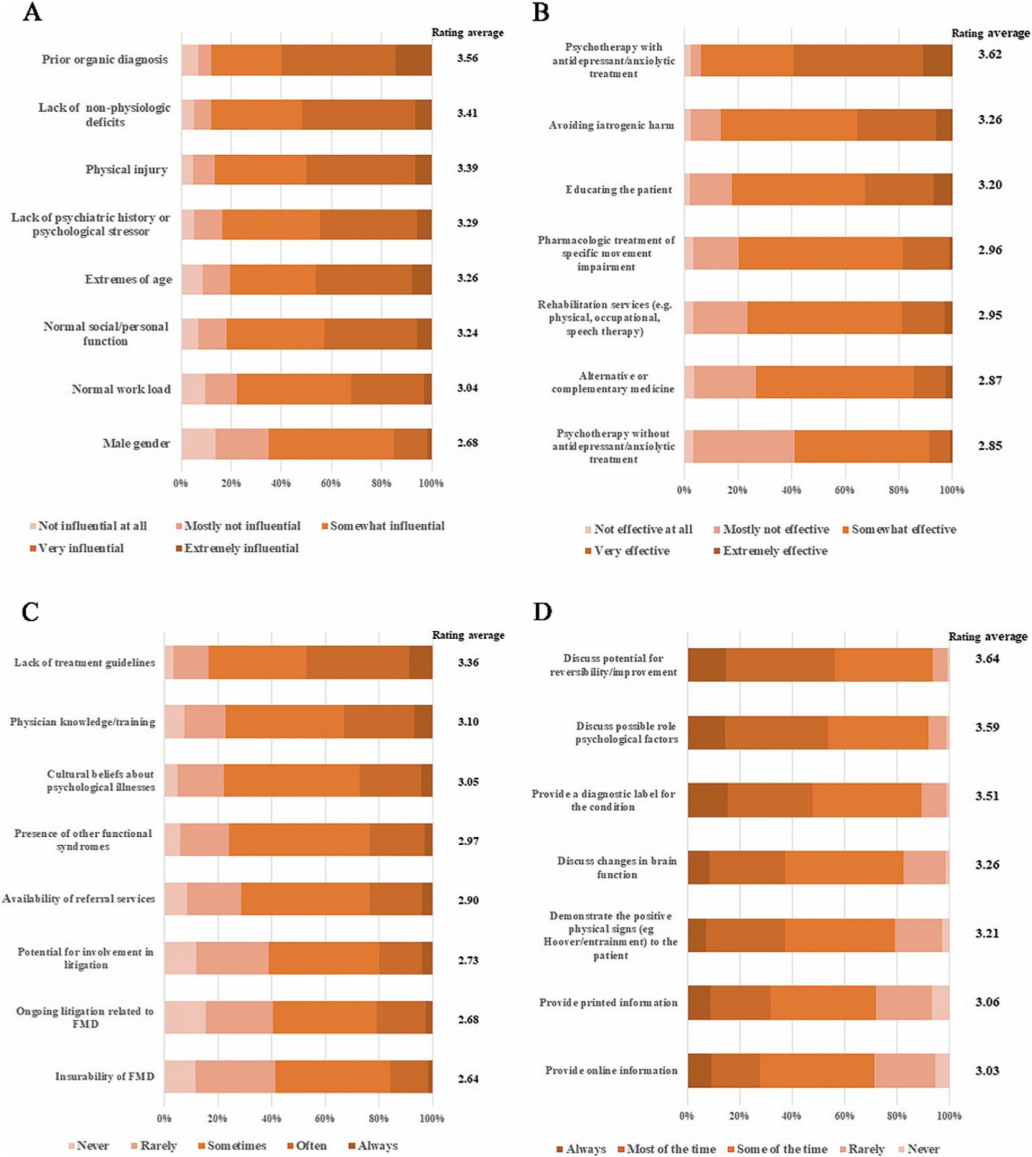
Opinions and clinical practice about diagnosing and managing FMD. Catalogues were listed in descending order by average ratings. Intensity of color indicates the extent of agreement (dark orange) or disagreement (light orange). **A** Influence of predictors for non-FMD diagnosis; **B** Effectiveness of treatment strategies of FMD; **C** Restrictions in managing patients with FMD; **D** Educating the patient about FMD diagnosis

Over half believed that prior diagnosis of an organic disorder provided by a reliable neurologist (59.9%), lack of associated non-physiologic deficits (51.8%), and evidence of physical injury (50.0%) were ‘very influential’ or ‘extremely influential’ for a non-PMD diagnosis. Meanwhile, gender seemed not important for diagnosis (Fig. 2A). Normal social or personal function was associated with normal work load (*r* = 0.564, *p* < 0.01), lack of psychiatric history or psychological stressor (*r* = 0.449, *p* < 0.01). Evidence of physical injury was correlated to lack of non-physiologic deficits (*r* = 0.506, *p* < 0.01), and lack of psychiatric history or psychological stressor (*r* = 0.411, *p* < 0.01).

### Management

A majority (77.4%) of the respondents may refer patients to a neuropsychiatrist or psychiatrist experienced in FMD, followed by psychologist or psychotherapist experienced in FMD (53.2%) (Fig. S4). Few respondents reported that their original diagnosis of FMD was questioned by psychiatrists, psychologists, rehabilitation specialists often (4.4%) or always (0.7%). A minority of respondents paid more attention on the treatment of FMD than on that of organic disorders. Respondents tended to prioritize treatment of symptoms that mainly led to disability, regardless whether they were thought organic or functional (52.3%). About 30% of them put diagnosis and treatment organic disorder in the first place.

Opinions on effectiveness of various treatment strategies differed. Taken together, psychotherapy with antidepressant/anxiolytic treatment was believed to be the most effective as nearly 60% respondents rated it as ‘very effective’ or ‘extremely effective’. Effectiveness of avoiding iatrogenic harm and educating the patient were ranked second and third, respectively, and in high association (*r* = 0.702, *p* < 0.01). By contrast, psychotherapy without antidepressant/anxiolytic treatment was thought less effective than other treatment strategies (Fig. 2B). When educating a patient about the FMD diagnosis, the respondents mostly discussed potential for reversibility/improvement and possible psychological factors (Fig. 2D).

Management ability of clinicians was generally (‘often’ or ‘always’) restricted by lack of treatment guidelines (47.2%), and less related knowledge and training (37.9%). The two limitations were related to each other (*r* = 0.570, *p* < 0.01). Insurability and litigation of FMD were deemed as limitations less frequently (Fig. 2C).

### Predictors of prognosis

**Fig. 3:**
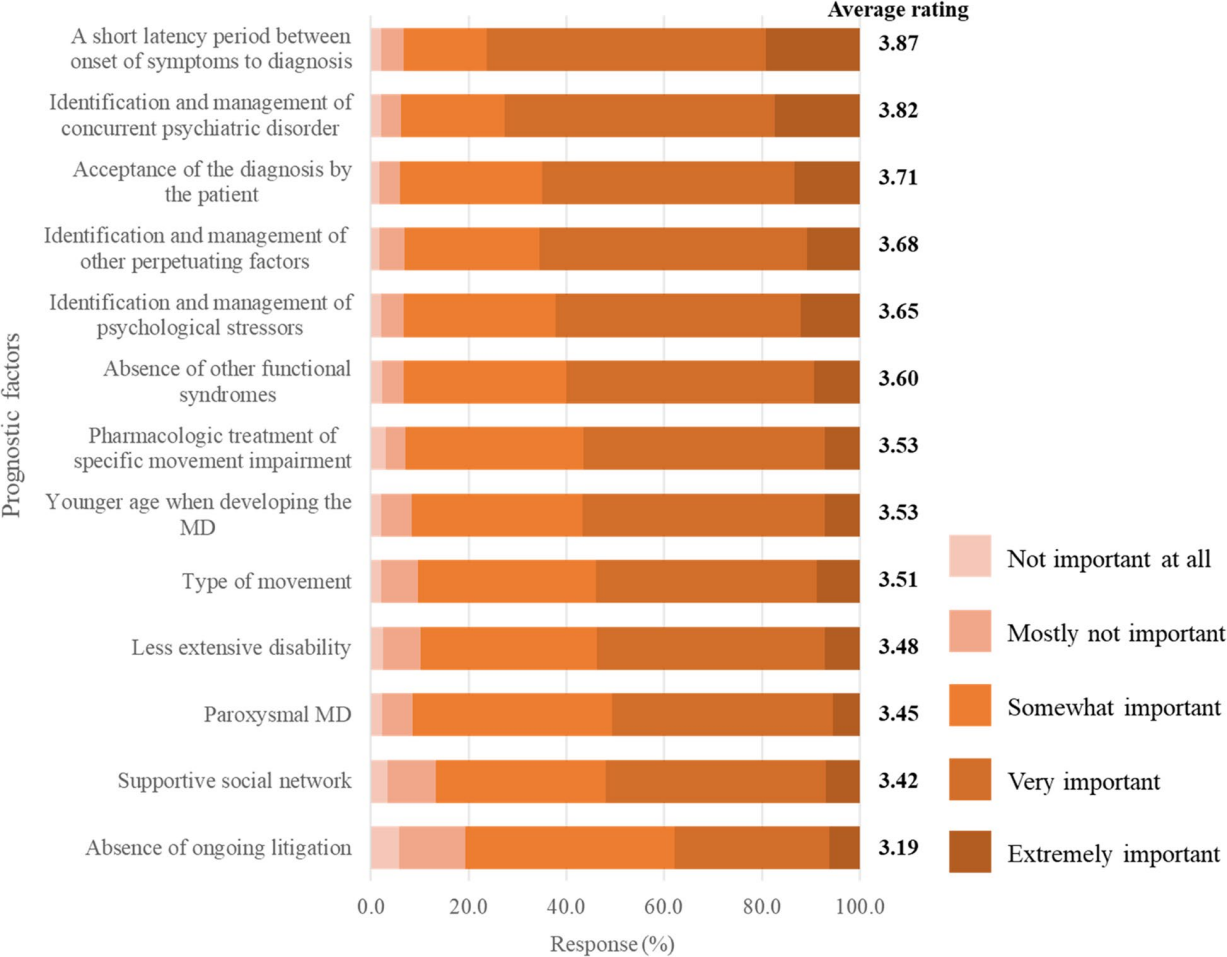
Importance of predictors for a better prognosis of FMD. Catalogues were listed in descending order by average ratings

Early diagnosis of FMD, identification and management of concurrent psychiatric disorder, and acceptance of the diagnosis by the patient played important roles in a better prognosis (Fig. 3). In the contrast, lack of ongoing litigation, supportive social network, and paroxysmal type might be minor predictors.

### Terminology and other concerns

**Fig. 4:**
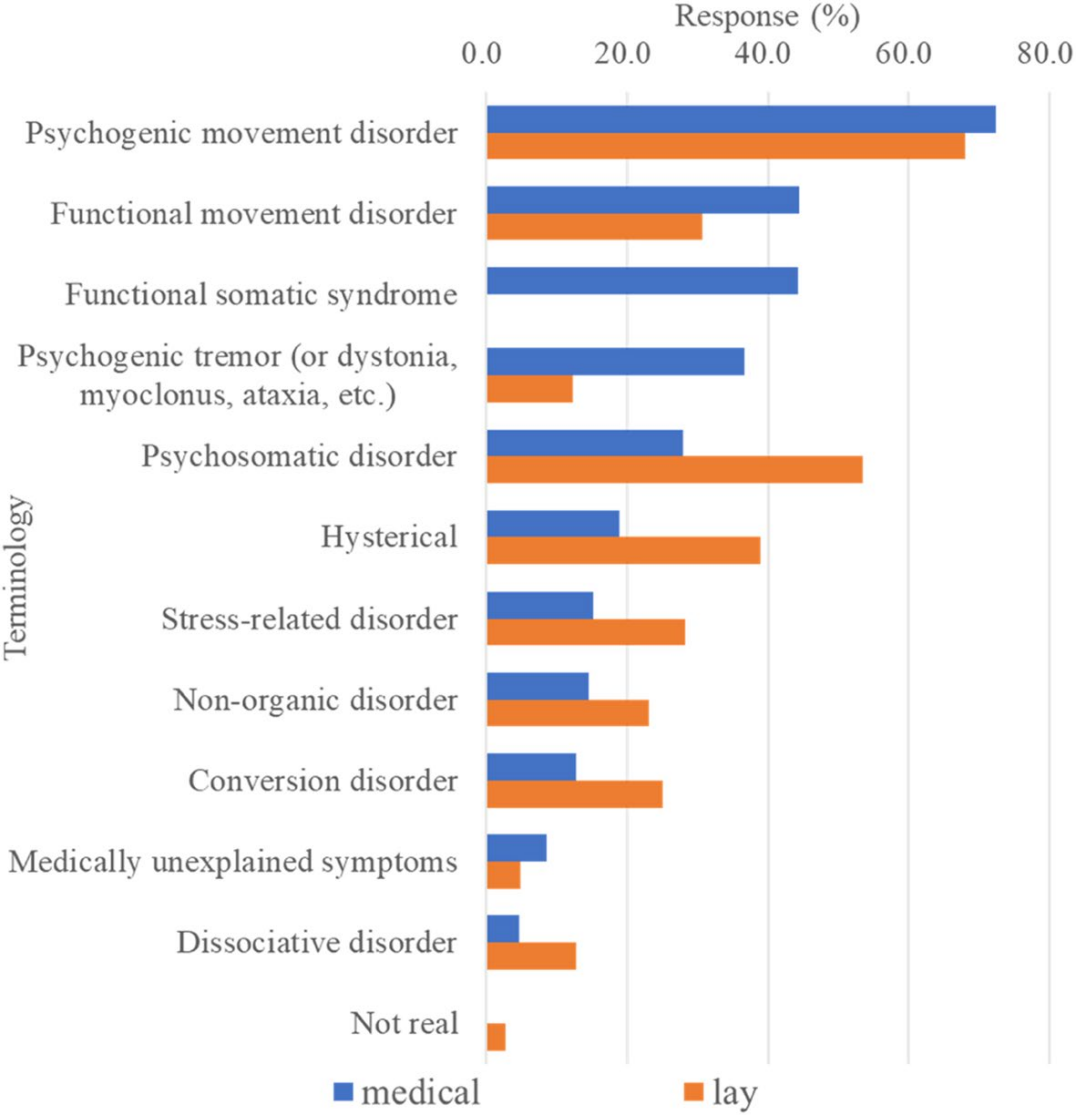
Terms used in medical communications and lay public. The preferred terms in communicating with medical professionals (blue) and lay public (orange) when respondents were asked to select the top three

The most common terminology remained ‘psychogenic movement disorder’ in the present study. Additionally, ‘functional movement disorder’ and ‘functional somatic syndrome’ were also endorsed in experts’ communication. ‘Psychosomatic disorder’ was often used as lay term. Other terms are illustrated in Fig. 4. Among all respondents, 16.4% (71/434) answered the open-ended question about other unsolved issues important in FMD (Table S2).

## Discussion

The present investigation revealed a clear picture of current views of FMD, and found that opinions and clinical practice of FMD varied widely among Chinese clinicians for the first time. This could be partially explained by lack of diagnosis/treatment guidelines and physician knowledge, which were also taken as important limitations in management of FMD. Meanwhile, both general neurologists and subspecialists of MDs were recruited in this survey. Considering that the practitioners with more experience in MD reported more FMD patients assessed monthly (Table 2), it is supposed that these practitioners were more familiar with diagnosis of FMD.

Compared to the latest survey conducted in members of International MDS (summarized in Table 3) [8], there were differences in results of the present survey. Despite the term having shifted to ‘Functional movement disorders’ in recent years, the Chinese expression of ‘psychogenic movement disorders’ was endorsed by most clinicians. However, opinions and clinical approaches differed in diagnosis, treatment, and prognosis. The selective bias should be taken into consideration. Most respondents investigated in the survey of International MDS were academic clinicians/researchers interested in FMD, and were fellowship trained in subspecialty of MDs. Whereas more general neurologists were surveyed in the present survey. Furthermore, different practice patterns could not be ignored as indicated in the previous surveys [7, 8].

**Table 3:** Differences between results of this Chinese survey and that of recent MDS survey (percentages of respondents)

Descriptions	Chinese survey	Recent MDS survey (2018)
More than 3 FMD patients seen monthly	18.5%	34%
Identifying a comorbid organic neurological disorder ‘sometimes’ or ‘frequently’	37.7%	41%
Concerned about missing another organic diagnosis in FMD patients	55.7%	64%
Role and responsibility (more than providing a diagnosis)	94.5%	99.1%
Disliking seeing FMD patients	24.7%	29%
Necessary for clinically definite FMD (top three)		
Incongruent MD	84.8%	60.7%
Multiple somatizations	82.3%	
Emotional disturbance	80.9%	
Functional signs		78.1%
Inconsistent over time		51.6%
Use suggestion to assist with diagnosis	29.5%	43.1%
Use placebo to assist with diagnosis	17.7%	8.8%
Request neurological investigations before diagnosing	58.1%	47%
Electrophysiology for confirmation	89.8%	60%
Discuss results of the electrophysiology testing with patients (‘often’ and ‘always’)	29.1%	21.2%
Non-FMD diagnosis indicators (top three)		
A prior organic diagnosis	60%	43%
Lack of non-physiologic deficits	52%	
Physical injury	50%	37%
Extremes of age		36%
Refer patients		
Neuropsychiatrist or psychiatrist experienced in FMD	77.4%	
General Psychiatrist		56%
Very and extremely effective treatments (top three)		
Psychotherapy with antidepressant/ anxiolytic treatment	59.2%	
Avoiding iatrogenic harm	35.5%	58%
Educating the patient	32.7%	53%
Rehabilitation services		40%
Limitations in managing patients		
Lack of treatment guidelines	47.3%	39%
Physician knowledge/training	32.9%	
Cultural beliefs about psychological illnesses	27.2%	50%
Availability of referral services		48%
Important for indicating a better prognosis (top three)		
Early diagnosis of FMD	76.2%	
Identification and management of concurrent psychiatric disorder	72.6%	About 85%
Acceptance of the diagnosis by the patient	65.0%	More than 90%
Identification and management of psychological stressors		About 84%
Educating a patient (‘most of the time’ and ‘always’)		
Discuss potential for reversibility/improvement	56.2%	90%
Discuss possible role psychological factors	53.7%	85%

Note: The blank cells indicate the differences of top options between the two surveys. For instance, incongruent MD, multiple somatizations, and emotional disturbance ranked top three necessary for Chinese neurologist in opinions of clinically definite FMD, whereas incongruent MD, functional signs, and inconsistent over time were endorsed by most MDS members

Despite some significant differences between the present study and the previous MDS study, some points of view were similar. For instance, more than half of the respondents worried about the potential of missing another organic disorder in patients with FMD. Additionally, the respondents tended to believe that an incongruent movement disorder was necessary/essential for clinically definite FMD. In terms of indicating non-FMD diagnosis, a prior organic diagnosis and physical injury were endorsed by both sides. Avoiding iatrogenic harm and educating the patient were considered very or extremely effective in treating patients with FMD by practitioners at home and abroad. The clinicians’ ability in managing FMD patients was often restricted by lack of treatment guidelines and cultural beliefs about psychological illnesses. Identification and treatment of concurrent psychiatric disorder and acceptance of the diagnosis by the patient were crucial for predicting a better prognosis. Regarding patient education, discussion about potential for reversibility/improvement and possible psychological factors were usually adopted.

### Challenges in diagnosis

A great proportion of respondents put emphasis on the presence of multiple somatizations and emotional disturbance in diagnosing a definite FMD. This might indicate a misunderstanding of FMD since the fifth version of Diagnostic and Statistical Manual of Mental Disorders (DSM-5) indicated the importance of making a ‘positive’ diagnosis rather than emphasizing precipitating stressors [6]. Neurological examination which demonstrates inconsistency and/or incongruence can be essential to establish a positive diagnosis [9]. Additionally, only 9% of the practitioners informed the patients of certain diagnosis during initial assessment and did not request standard neurological investigations while nearly 75% requested investigations before or not informing patients of the diagnosis. Based on this evidence, it is inferred that only few of the respondents were good at diagnosing FMD. It is also reflected by the number of patients with FMD assessed per month in clinics by the respondents. A considerable proportion of respondents reported less than 3 or were uncertain about it.

Electrophysiology testing was more widely used in the present survey. In this condition, Chinese clinicians explained the results of electrophysiological testing to patients a little more often. It was also reported that electrophysiological testing was more commonly applied outside the USA. Electrophysiology testing proved useful in supporting diagnosis of FMD, especially subtype of tremor. But for cases with long-term or mixed with organic disorder, it might not be supportive [10].

A substantial proportion of respondents were concerned about missing a potential organic diagnosis in the both present survey (55.7%) and previous survey (64%). In fact, a meta-analysis indicated that the rate of misdiagnosis of conversion symptoms had declined to about 4% since 1970 [11]. It seems that this level of worry is unnecessary.

### Challenges in management

The results showed different referral preferences (Neuropsychiatrist or psychiatrist focused on FMD vs General psychiatrist) between Chinese clinicians and international MDS members. Considering the treatment of FMD, psychotherapy with antidepressant/ anxiolytic treatment was rated the most effective by Chinese clinicians, which differed from the survey conducted by MDS where avoiding iatrogenic harm was endorsed by most respondents. The related favorable evidence was sparse. One small-sized clinical trial found that patients with primary conversion symptoms and with depression or anxiety may respond to antidepressant drugs [12]. Other previous research suggested some treatment strategies that might be effective. For example, physical therapy might contribute to improvement of symptoms [13–15]. Physiotherapy was recommended in definite FMD patients who desired improvement and serve as the foundation of psychological treatment. The related consensus emphasized the importance of physiotherapy and occupational therapy in the multidisciplinary management of patients with FMD [16, 17]. Therefore, rehabilitation services played a key role in the intervention of FMD, but they were ignored to some extent in clinical practice in China. In addition, another research found cognitive behavioral therapy (CBT) effective in alleviating specific motor symptoms, as well as depression and anxiety [18]. Contrarily, the adjunctive physical activity (APA) did not achieve satisfactory improvements [18]. The main disadvantages of these studies were the small size and recruitment of different types of movement disorder. Further studies should be conducted in large samples to verify the effectiveness of the therapies. Other potential treatments included multidisciplinary treatment, transcutaneous electrical stimulation and others [3].

Avoiding iatrogenic injury and patient education were emphasized again in this present survey. It was generally accepted that the treating process starts from delivering the diagnosis of PMD to the patient [3, 19]. Demonstrating the positive physical signs and talking with the patient about the potential for reversibility were highly appreciated, but a minority of the respondents explained the positive signs when educating the patient. Acceptance of the FMD diagnosis by the patient counted most in predicting a better prognosis in previous surveys [7]. However, most domestic clinicians were in favor of early diagnosis as predictor for a better prognosis. It was inconclusive but with a few supportive evidences [20]. Overall, outcomes of FMD seems unfavorable [20]**.**

### Limitations

Sampling bias existed as more neurologists with less experience in MDs subspecialty were recruited in this present survey. In this condition, the results might represent the general perceptions related to FMD in Chinese neurological clinicians. In addition, no-response bias also should be considered. The response rate was difficult to calculate because separate emails were not adopted to target respondents.

## Conclusions and recommendations

Opinions and clinical practice of Chinese practitioners not only varied among Chinese clinicians, but also differed from international peers. Chinese neurologists have insufficient knowledge of FMD, especially those with shorter clinical practice. FMD was generally neglected. We recommend to raise awareness of FMD in following aspects:

1. It is necessary to set up Chinese (translated or modified) version of the diagnostic criteria of FMD. Chinese diagnostic criteria may conduce to the clinical practice of neurologists.
2. A lack of treatment guidelines represents an important limitation in the management of patients with FMD. However, studies on effectiveness of treating FMD are scant in China. Combined efforts are needed to promote related research and establish guidelines.
3. It is necessary to decide whether to use ‘functional movement disorder’ instead of ‘psychological movement disorder’ in Chinese terminology, which would be in line with international standards.
4. FMD should be added to fellowship training and teaching period in China.
5. Except for FMD, the diagnosis/treatment of psychogenic nonepileptic seizures (PNES) or pseudo(−)seizure(s) are needed to be better understood as well in the future.

## Supplementary Information

**Additional file 1.**

**Additional file 2: Fig. S1.** Demographic information of the respondents. **Fig. S2.** Findings necessary for clinically definite diagnosis of FMD. **Fig. S3.** Suggestion or placebo used in documenting and diagnosing FMD. **Fig. S4.** Refer patients for treatment.

**Additional file 3: Table S1.** Affiliations of the External reviewers for the Chinese version of FMD questionnaire.

**Additional file 4: Table S2.** Other issues important in the diagnosis and treatment of FMD that have not been addressed (Answers of Item-21).

## Data Availability

The datasets generated and/or analysed during the current study are available from the corresponding author on reasonable request.

## Abbreviations

FMD: Functional movement disorder
PMD: Psychogenic movement disorder
MDS: Movement Disorder Society
MDs: Movement disorders
