# Supplementary material for: Opinions and clinical practice of functional movement disorders: a nationwide survey of clinicians in China
Source: BMC Neurol. 2021 Nov 9;21:435. doi: 10.1186/s12883-021-02474-4 (PMC8576952; doi:10.1186/s12883-021-02474-4)
Supplement: Supplementary file 1 — Additional file 1. [file 12883_2021_2474_MOESM1_ESM.docx]

**Opinions and Clinical Practice of Functional Movement Disorders: a nationwide survey of clinicians in China**

**Appendix**

**Expert opinions and clinical practices related to patients with** **functional (psychogenic) movement disorders**

Chinese version

Functional (Psychogenic) movement disorders are challenging to neurological, psychological and psychiatric clinicians in practice. Although clinicians have reached some agreement of diagnosis and disease management in the field of psychogenic movement disorders, the related sub-specialties have not been fully established in China. Additionally, the spread of such concepts in this field is limited and there are still many puzzles for many clinicians during practice. Follow the example of ***Movement Disorders Society***, herein, we sincerely invite you to take part in our survey anonymously. The questionnaire used in this survey is modified from that of ***Movement Disorders Society***, which would help us understand the character of Chinese clinicians on Functional movement disorders. Hereby, we hope to offer proposals about improving the management of functional movement disorders in China.

Wang Gang, PhD

Department of Neurology and Institute of Neurology

Rui Jin Hospital, Shanghai Jiao Tong University School of Medicine

And the sequence of each question was adjusted to fit in Chinese culture.

**I. DIAGNOSIS**

1. **Which of the following findings do you believe are *essential* or *absolutely necessary* for a clinically definite diagnosis of Functional (Psychogenic) Movement Disorders (FMD)? (check all that apply)**

__ A. Evidence of an emotional disturbance (e.g., mood fluctuations)

__ B. Presence of an obvious psychiatric disturbance (e.g., major depression, PTSD)

__ C. Presence of multiple somatizations

__ D. Presence of functional (psychogenic) signs on neurological examination

__ E. Movement disorder is incongruent with a classical movement disorder

__ F. Movement disorder is inconsistent over time

1. **How often do you use suggestion (guiding or encouraging a change in the movement) to document and diagnose FMD?**

**__** A. Never

**__** B. Rarely

**__** C. Sometimes

**__** D. Often

**__** E. Always

1. **How often do you use a placebo (resolution or worsening of a movement with an inert intravenous or oral drug) to document and diagnose FMD?**

**__** A. Never

**__** B. Rarely

**__** C. Sometimes

**__** D. Often

**__** E. Always

**4. If a patient has unequivocal clinical features incompatible with an organic movement disorder with no features suggesting another underlying neurological problem condition which of the following best describes the approach you most often use?**

__ A. I inform the patient that they have a clinically definite FMD diagnosis during my initial assessment and I do not request standard neurological investigations (e.g., brain MRI, head CT, EEG, evoked potentials, etc)

__ B. Ask the patient to return for a reexamination at a follow-up visit after which I inform the patient of the FMD diagnosis

__ C. I request standard neurological investigations to rule out organic causes after which I inform the patient of the FMD diagnosis

__ D. I request standard neurological investigations after which I do not inform the patient of the FMD diagnosis or psychogenic causation

__ E. Recommend that a second neurology consultation with another movement disorders expert be obtained before I disclose the diagnosis to the patient

**5. In which cases do you use electrophysiology testing to confirm the myoclonus or tremor forms of FMD (e.g., ascertainment of Bereitschftspotential and latency to movement onset for myoclonus, entrainment or change in frequency for tremor, etc.)?**

__ A. To confirm the diagnosis in all cases

__ B. To confirm the diagnosis in selected cases only when clinical examination alone is insufficient to establish the diagnosis (i.e., uncertain cases)

__ C. To confirm the diagnosis in uncertain cases AND selected cases of “clinically definite” FMD

__ D. I do not have access to an electrophysiology laboratory to do testing to confirm the diagnosis of FMD

__ E. I do not think electrophysiology is useful for diagnosing FMD

**6. How often do you use the results of the electrophysiology testing to explain the diagnosis to the patient?**

__ A. Never

__ B. Rarely

__ C. Sometimes

__ D. Often

__ E. Always

__ F. Not applicable (no access)

**7. How influential are each of the following for indicating a diagnosis other than FMD?**

|  | Not influential at all | Mostly not influential | Somewhat influential | Very influential | Extremely influential |
| --- | --- | --- | --- | --- | --- |
| Extremes of age (i.e., <6 years, >75 years) | Ο | Ο | Ο | Ο | Ο |
| Male gender | Ο | Ο | Ο | Ο | Ο |
| Prior diagnosis of an organic disorder by an experienced/ reliable neurologist | Ο | Ο | Ο | Ο | Ο |
| Normal social or personal function | Ο | Ο | Ο | Ο | Ο |
| Normal work load with little or no employment disruption | Ο | Ο | Ο | Ο | Ο |
| Lack of associated non-physiologic deficits | Ο | Ο | Ο | Ο | Ο |
| Lack of psychiatric history or psychological stressor | Ο | Ο | Ο | Ο | Ο |
| Evidence of physical injury | Ο | Ο | Ο | Ο | Ο |

**8. How often do you find an underlying organic neurologic disorder (e.g., multiple sclerosis) in your patients with FMD? (This question relates to potential comorbidities, not misdiagnosis).**

__ A. Never

__ B. Very rarely

__ C. Rarely

__ D. Sometimes

__ E. Frequently

**9. To what extent are you concerned about potentially missing another organic disorder in FMD patients?**

__ A. Very concerned

__ B. Somewhat more concerned compared to other disorders I manage

__ C. Neither more or less concerned compared to other disorders I manage

__ D. Somewhat less concerned compared to other disorders I manage

__ E. Not at all concerned

**10. In cases with a dominant clinically definite FMD where you suspect an underlying “organic” disorder, you:**

__ A. Focus the diagnosis and treatment on the organic disorder, and not diagnose a concurrent FMD

__ B. Determine the major source of disability and prioritize treatment of that symptom, regardless of whether it is organic or functional

__ C. Treat the FMD first and treating the organic movement disorder only if it is contributing to disability once the FMD is better managed

__ D. Treat the FMD and if only if this is unsuccessful then try to treat the organic movement disorder

**II. TREATMENT**

**11. To which of the following can you ~~readily~~ refer patients for treatment? (check all that are available to you)**

__ A. General Psychiatrist

__ B. Neuropsychiatrist or psychiatrist experienced in FMD

__ C. General psychologist or psychotherapist

__ D. Psychologist experienced in FMD

__ E. Rehabilitation or physiotherapy specialist

__ F. Psychiatric inpatient services

__ G. Inpatient multidisciplinary rehabilitation

__ H. Other specialty clinic: __________________________

__ I. I have no access to the above services

**12. In your opinion, how effective are each of the following for treating FMD?**

|  | Not effective at all | Mostly not effective | Somewhat effective | Very effective | Extremely effective |
| --- | --- | --- | --- | --- | --- |
| Pharmacologic treatment of specific movement impairment | Ο | Ο | Ο | Ο | Ο |
| Psychotherapy with antidepressant/anxiolytic treatment | Ο | Ο | Ο | Ο | Ο |
| Psychotherapy *without* antidepressant/anxiolytic treatment | Ο | Ο | Ο | Ο | Ο |
| Rehabilitation services (e.g. physical, occupational, speech therapy) | Ο | Ο | Ο | Ο | Ο |
| Alternative or complementary medicine | Ο | Ο | Ο | Ο | Ο |
| Educating the patient | Ο | Ο | Ο | Ο | Ο |
| Avoiding iatrogenic harm | Ο | Ο | Ο | Ο | Ο |

**13. How often does each of the following limit your ability to manage patients with FMD?**

|  | Never | Rarely | Sometimes | Often | Always |
| --- | --- | --- | --- | --- | --- |
| Physician knowledge/training | Ο | Ο | Ο | Ο | Ο |
| Lack of treatment guidelines | Ο | Ο | Ο | Ο | Ο |
| Insurability of FMD | Ο | Ο | Ο | Ο | Ο |
| Availability of referral services | Ο | Ο | Ο | Ο | Ο |
| Cultural beliefs about psychological illnesses | Ο | Ο | Ο | Ο | Ο |
| Ongoing litigation related to the FMD | Ο | Ο | Ο | Ο | Ο |
| Potential for your involvement in litigation | Ο | Ο | Ο | Ο | Ο |
| Presence of other functional syndromes (e.g., fibromyalgia, irritable bowel syndrome, others) | Ο | Ο | Ο | Ο | Ο |

**14. How frequently do psychiatrists, psychologists, or rehabilitation specialists question your original diagnosis and recommend you to reconsider the neurological basis for the disorder?**

**__** A. Never

**__** B. Rarely

**__** C. Sometimes

**__** D. Often

**__** E. Always

**15. What do you think your role or responsibility is in assessing FMD?**

**__** A. To provide only a diagnosis

**__** B. To provide a diagnosis and attempt to secure expert management (e.g. psychiatry, physical and occupational therapy)

**__** C. To provide a diagnosis and coordinate interdisciplinary long-term management

**__** D. To provide a diagnosis and manage the patient’s care personally

**III. PROGNOSIS**

**16. How important are each of the following for a better prognosis of FMD?**

|  | Not important at all | Mostly not important | Somewhat important | Very important | Extremely important |
| --- | --- | --- | --- | --- | --- |
| A short latency period between onset of symptoms to diagnosis | Ο | Ο | Ο | Ο | Ο |
| Younger age when developing the movement disorder | Ο | Ο | Ο | Ο | Ο |
| Less extensive disability | Ο | Ο | Ο | Ο | Ο |
| Type of movement (e.g., tremor or chorea vs. dystonia or ataxia) | Ο | Ο | Ο | Ο | Ο |
| Paroxysmal rather than persistent or fixed movement disorders | Ο | Ο | Ο | Ο | Ο |
| Absence of ongoing litigation | Ο | Ο | Ο | Ο | Ο |
| Supportive social network | Ο | Ο | Ο | Ο | Ο |
| Identification and management of psychological stressors | Ο | Ο | Ο | Ο | Ο |
| Identification and management of concurrent psychiatric disorder | Ο | Ο | Ο | Ο | Ο |
| Identification and management of other perpetuating factors (e.g. financial difficulties) | Ο | Ο | Ο | Ο | Ο |
| Absence of other functional syndromes (e.g., fibromyalgia, irritable bowel syndrome, others) | Ο | Ο | Ο | Ο | Ο |
| Pharmacologic treatment of specific movement impairment | Ο | Ο | Ο | Ο | Ο |
| Acceptance of the diagnosis by the patient | Ο | Ο | Ο | Ο | Ο |

**IV. TERMINOLOGY**

**17. Choose the top three *medical terms* you think should be used when *experts communicate* with each other about FMD (Choose the top 3)**

| Psychogenic movement disorder | Ο |
| --- | --- |
| Psychogenic tremor (or dystonia, myoclonus, ataxia, etc.) | Ο |
| Functional somatic syndrome | Ο |
| Medically unexplained symptoms | Ο |
| Functional movement disorder | Ο |
| Psychosomatic disorder | Ο |
| Stress-related disorder | Ο |
| Hysterical | Ο |
| Non-organic disorder | Ο |
| Dissociative disorder |  |
| Conversion disorder | Ο |

**18. Choose the top three *lay terms* you think should be used *when experts communicating with patients or the lay public* about FMD (Choose the top 3)**

|  | Top 3 |
| --- | --- |
| Psychogenic movement disorder | Ο |
| Psychogenic tremor (or dystonia, myoclonus, ataxia, etc.) | Ο |
| Stress-related disorder | Ο |
| Medically unexplained symptoms | Ο |
| Functional movement disorder | Ο |
| Psychosomatic disorder | Ο |
| Conversion disorder | Ο |
| Dissociative disorder | Ο |
| Non-organic disorder | Ο |
| Hysteria | Ο |
| Not real | Ο |

**19. How often do you perform the following when educating a patient about the diagnosis of FMD?**

|  | Always | Most of the time | Some of the time | Rarely | Never |
| --- | --- | --- | --- | --- | --- |
| Provide a diagnostic label for the condition | Ο | Ο | Ο | Ο | Ο |
| Demonstrate the positive physical signs (eg Hoover/entrainment) to the patient | Ο | Ο | Ο | Ο | Ο |
| Discuss changes in brain function | Ο | Ο | Ο | Ο | Ο |
| Discuss possible role psychological factors | Ο | Ο | Ο | Ο | Ο |
| Discuss potential for reversibility/improvement | Ο | Ο | Ο | Ο | Ο |
| Provide printed information (ie something for the patient to physically take away) | Ο | Ο | Ο | Ο | Ο |
| Provide online information | Ο | Ο | Ο | Ο | Ο |

**20. Compared to patients with other movement disorders such as Parkinson’s disease, how would you rank your personal preference in taking care of patients with FMD?**

**__** A. I very much look forward to seeing patients with FMD in my clinic

**__** B. I somewhat look forward to seeing patients with FMD in my clinic

**__** C. I am neither looking forward to nor dislike seeing patients with FMD in my clinic

**__** D. I somewhat dislike seeing patients with FMD in my clinic

**__** E. I very much dislike seeing patients with FMD in my clinic

**21. Additional Comments: Are there any other issues that you believe are important in the diagnosis and treatment of FMDs that have not been addressed?**

**V. DEMOGRAPHIC INFORMATION**

Please answer the following questions about you and your practice

**1. Sex**

[ ] Male

[ ] Female

**2. Age (years)**

[ ] 25-35 [ ] 36-45 [ ] 46-55 [ ] 56-65 [ ] >66

**3. Grade of the hospital in clinical practice**

[ ] Grade III [ ] Grade II [ ] Grade I

**4. Professional title**

[ ] resident doctor

[ ] visiting staff

[ ] associate chief physician

[ ] chief physician

**5. Length of fellowship training in movement disorders**

[ ] None [ ] 1 year [ ] 2 years [ ] 3 years [ ] 4 years

**6. Years in practice? (Post-residency)**

[ ] < 5 years [ ] 6-10 years [ ] 11-15 years [ ] 16-20 years [ ] >21 years

**7. Practice field (Department)**

[ ] Neurology

[ ] Specialty of MOVEMENT DISORDER

[ ] Physician

[ ] Psychiatry or Psychology

[ ] Others

**8. Where is your practice?**

Location (city-province) [ Drop down menu]

**9. Which best describes the number of patients with PMD that you see per month?**

[ ] <1 [ ] 1-3 [ ] 4-6 [ ] 7-10 [ ] >11 [ ] Uncertain

**10. Which best describes the number of patients with all movement disorders you see per month?**

[ ] <30 [ ] 31-45 [ ] 46-60 [ ] 61-80 [ ] >80 [ ] Uncertain
