# Supplementary material for: Opinions and clinical practice of functional movement disorders: a nationwide survey of clinicians in China
Source: BMC Neurol. 2021 Nov 9;21:435. doi: 10.1186/s12883-021-02474-4 (PMC8576952; doi:10.1186/s12883-021-02474-4)
Supplement: Supplementary file 2 — Additional file 2: Fig. S1. Demographic information of the respondents. Fig. S2. Findings necessary for clinically definite diagnosis of FMD. Fig. S3. Suggestion or placebo used in documenting and diagnosing FMD. Fig. S4. Refer patients for treatment. [file 12883_2021_2474_MOESM2_ESM.docx]

**Fig.S1: Demographic information of the respondents**

**Fig. S2: Findings necessary for clinically definite diagnosis of FMD**

**Fig. S3：Suggestion or placebo used in documenting and diagnosing FMD**

**Fig. S4: Refer patients for treatment**
