# Supplementary material for: Opinions and clinical practice of functional movement disorders: a nationwide survey of clinicians in China
Source: BMC Neurol. 2021 Nov 9;21:435. doi: 10.1186/s12883-021-02474-4 (PMC8576952; doi:10.1186/s12883-021-02474-4)
Supplement: Supplementary file 3 — Additional file 3: Table S1. Affiliations of the External reviewers for the Chinese version of FMD questionnaire. [file 12883_2021_2474_MOESM3_ESM.docx]

| No. | Department | Institution |
| --- | --- | --- |
| 1 | Department of Neurology | First Affiliated Hospital of Anhui Medical University |
| 2 | Department of Neurology | Shandong Province Hospital |
| 3 | Department of Neurology | Cangzhou Hospital of Integrated Traditional Chinese and Western of Hebei Province |
| 4 | Department of Neurology | West China Hospital affiliated to Sichuan University |
| 5 | Department of Psychiatry | West China Hospital affiliated to Sichuan University |
| 6 | Human Motor Control Section, Medical Neurology Branch | National Institute of Neurological Disorders and Stroke, National Institutes of Health |
| 7 | Department of Neurology | Peking Union Medical College Hospital |
| 8 | Department of Neurology | The First People's Hospital of Foshan |
| 9 | Department of Neurology | Guangdong General Hospital |

**Table S1: Affiliations of the External reviewers for the Chinese version of FMD questionnaire**

Statement: All subjects were informed and agreed the publication of identifying information in an online open-access publication.
