# Supplementary material for: Opinions and clinical practice of functional movement disorders: a nationwide survey of clinicians in China
Source: BMC Neurol. 2021 Nov 9;21:435. doi: 10.1186/s12883-021-02474-4 (PMC8576952; doi:10.1186/s12883-021-02474-4)
Supplement: Supplementary file 4 — Additional file 4: Table S2. Other issues important in the diagnosis and treatment of FMD that have not been addressed (Answers of Item-21). [file 12883_2021_2474_MOESM4_ESM.docx]

**Table S2: Other issues important in the diagnosis and treatment of FMD that have not been addressed (Answers of Item-21)**

| Themes | Summarized descriptions |
| --- | --- |
| **Diagnosis** | Have difficulty in clinical diagnosis of FMD |
|  | Diagnosis and follow-up |
|  | Organic movement disorders combined with FMD |
|  | Features of electrophysiological testing on FMD |
|  | Assessment methods |
|  | Lack of diagnostic biomarkers |
| **Role of neurologist** | Who is responsible for diagnosis and treatment? |
|  | Can neurologists diagnose psychological illness? |
|  | Beyond authority of neurologists whether or not |
| **Guidelines** | Lack of Chinese guidelines on diagnosis or treatment |
|  | Lack of expert consensus |
| **Training** | Lack of experience in FMD |
|  | Look forward to related training |
|  | Lack of systematic knowledge of effective treatment strategies |
|  | Training about tremor analysis |
|  | Make more clinicians familiar with FMD by popularization of relevant knowledge |
| **Multidisciplinary cooperation** | Multidisciplinary consultation |
|  | Multidisciplinary referral |
|  | Multidisciplinary treatment |
|  | Improve awareness of multidisciplinary clinicians about FMD |
| **Treatment** | How effective of the treatments? |
|  | Standard psychotherapy |
|  | Long-term management |
| **Education** | Acceptance by the patient |
|  | How to educating the patient? |
|  | Lack of public attention |
|  | Popularization of FMD |
| **Research** | Pathogenic mechanisms and physiological (biochemical) basis |
|  | Brain function tests |
